# Halide Homogenization for High-Performance Blue Perovskite Electroluminescence

**DOI:** 10.34133/2020/9017871

**Published:** 2020-12-24

**Authors:** Lu Cheng, Chang Yi, Yunfang Tong, Lin Zhu, Gunnar Kusch, Xiaoyu Wang, Xinjiang Wang, Tao Jiang, Hao Zhang, Ju Zhang, Chen Xue, Hong Chen, Wenjie Xu, Dawei Liu, Rachel A. Oliver, Richard H. Friend, Lijun Zhang, Nana Wang, Wei Huang, Jianpu Wang

**Affiliations:** ^1^Key Laboratory of Flexible Electronics (KLOFE) & Institute of Advanced Materials (IAM), Nanjing Tech University (NanjingTech), 30 South Puzhu Road, Nanjing 211816, China; ^2^Department of Materials Science and Metallurgy, University of Cambridge, 27 Charles Babbage Road, Cambridge CB3 0FS, UK; ^3^State Key Laboratory of Integrated Optoelectronics, Key Laboratory of Automobile Materials of MOE and College of Materials Science and Engineering, Jilin University, Changchun 130012, China; ^4^Frontiers Science Center for Flexible Electronics (FSCFE) & Shaanxi Institute of Flexible Electronics (SIFE), Northwestern Polytechnical University (NPU), 127 West Youyi Road, Xi'an 710072, China; ^5^Cavendish Laboratory, University of Cambridge, JJ Thomson Avenue, Cambridge CB3 0HE, UK

## Abstract

Metal halide perovskite light-emitting diodes (LEDs) have achieved great progress in recent years. However, bright and spectrally stable blue perovskite LED remains a significant challenge. Three-dimensional mixed-halide perovskites have potential to achieve high brightness electroluminescence, but their emission spectra are unstable as a result of halide phase separation. Here, we reveal that there is already heterogeneous distribution of halides in the as-deposited perovskite films, which can trace back to the nonuniform mixture of halides in the precursors. By simply introducing cationic surfactants to improve the homogeneity of the halides in the precursor solution, we can overcome the phase segregation issue and obtain spectrally stable single-phase blue-emitting perovskites. We demonstrate efficient blue perovskite LEDs with high brightness, e.g., luminous efficacy of 4.7, 2.9, and 0.4 lm W^−1^ and luminance of over 37,000, 9,300, and 1,300 cd m^−2^ for sky blue, blue, and deep blue with Commission Internationale de l'Eclairage (CIE) coordinates of (0.068, 0.268), (0.091, 0.165), and (0.129, 0.061), respectively, suggesting real promise of perovskites for LED applications.

## 1. Introduction

Low-temperature solution-processed halide perovskite LEDs, which can present high brightness and efficient electroluminescence (EL) with good colour purity [1, 2], show great application potential in lighting and displays. In particular, perovskite LEDs can be an important technology for emerging displays, such as augmented and virtual reality displays, where usually a brightness of over 10,000 cd m^−2^ is required in pixel areas [3]. So far, high efficiency EL at very high brightness has been demonstrated in near-infrared and green perovskite LEDs [4–6]. However, spectrally stable blue perovskite LEDs with high brightness are difficult to achieve. In principle, there are two approaches to tune the bandgap of the perovskite to blue emission. The first is to reduce the dimensionality of lead bromide-based perovskites and exploit the quantum confinement effect (e.g., quasi-two-dimensional or quantum-dot perovskites) [7, 8]. Blue EL with high peak EQE has been demonstrated based on low-dimensional perovskites [9, 10], though the devices suffer from low brightness due to the poor charge transport and serious efficiency roll-off at high current density caused by nonradiative Auger recombination [11]. For example, Liu et al. demonstrated efficient blue perovskite LEDs based on quasi-2D perovskites with embedded nanoparticles, but the peak EQE is at a low luminance of 54 cd m^−2^ and the maximum brightness is ~700 cd m^−2^ [9]. The second approach to achieve blue emission perovskites is through mixing Br and Cl in three-dimensional (3D) perovskites [12]. The 3D perovskites possess the merits of high charge mobility and low luminescence quenching at high excitations, which have been proven to be an effective strategy to achieve bright LEDs with high energy conversion efficiency [4, 6, 13]. However, phase segregation in mixed-halide 3D perovskites is generally found to prevent the achievement of spectrally stable LED devices [12]. Although the phase separation of mixed-halide perovskites has been observed earlier in the perovskite solar cell community, this critical issue is not well understood [14, 15]. Stable mixed-halide phases can only be observed with a small amount of Br in I- (or Cl in Br-) based 3D perovskites, and the stability falls off quickly as the ratio of the two halides gets closer [7, 15, 16]. Here, we reveal that the phase segregation issue can be effectively suppressed in homogeneous 3D CsPb (Br_1-*x*_Cl*_x_*)_3_ perovskites, even when at a 1 : 1 ratio of the two halides; this is achieved by using cationic additives to improve the mixing of Br and Cl in the precursor solution. Based on our 3D mixed-halide perovskites, we demonstrate efficient and spectrally stable LEDs with high brightness in all sky blue, blue, and deep blue areas.

## 2. Results

### 2.1. Bright and Spectrally Stable Blue Perovskite LEDs

**Figure 1:**
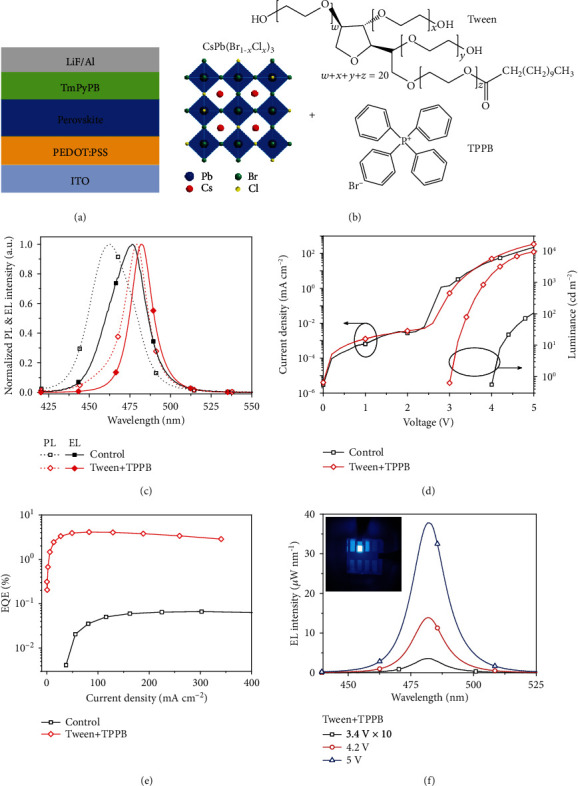
Characterization of perovskite LEDs without or with Tween-TPPB additives: (a) device structure; (b) composition of the emitting layer; (c) PL and EL spectra; (d) current density and luminance versus voltage; (e) EQE versus current density; (f) EL spectra upon various voltages of the Tween-TPPB-based device. Inset: photograph of the device.

The LED devices were fabricated with a structure of ITO/poly(3,4-ethylenedioxythiophene):poly(styrenesulfonate) (PEDOT:PSS, 30 nm)/perovskites (~50 nm)/1,3,5-tri(m-pyridin-3-ylphenyl)benzene (TmPyPB, 50 nm)/lithium fluoride (LiF, 1.2 nm)/aluminium (Al, 100 nm) (Figure 1(a)). A precursor solution of CsBr, PbBr_2_, and PbCl_2_ dissolved in a mixed solution of dimethyl sulfoxide (DMSO) and H_2_O (volume ratio, 0.92 : 0.08) was used to prepare the perovskite film, referred to as the control CsPb(Br_1-*x*_Cl*_x_*)_3_ films. The addition of H_2_O in the precursor solution can significantly increase the inclusion of Cl ions in the film [17], shifting the emission peak to the blue region (Figure S1). We introduce polyoxyethylene sorbitan monolaurate (Tween) and tetraphenylphosphonium bromide (TPPB) as additives in the precursor solution (Figure 1(b)) and will discuss their critical roles below.

The fabricated Tween-TPPB-based CsPb(Br_0.65_Cl_0.35_)_3_ device shows an EL peak at 482 nm and a full width at half maximum (FWHM) of 15 nm (Figure 1(c)), which is the narrowest reported blue emission (Table S1) [9, 10, 18, 19]. It has a CIE coordinate of (0.091, 0.165), at the edge of the blue emission [20]. Due to the good charge transport of 3D perovskites, the current density and EL intensity increase rapidly after the turn-on of LEDs (Figure 1(d)). We report a peak luminous efficacy of 2.9 lm W^−1^ and a luminance of 9,300 cd m^−2^ at a low voltage of 5 V, representing the highest brightness of blue perovskite LEDs (Table S1) [9, 10, 18]. The device reaches a peak EQE of 4.1% and shows low efficiency drop, maintaining a value of 3.4% even at a high luminance of >8,000 cd m^−2^ (Figure 1(e)). We note that this is the record efficiency of blue perovskite LEDs at high brightness [10]. The devices exhibit good reproducibility with an average peak EQE of 3.5% and a relative standard deviation of 9.3% (Figure S2(a)). Importantly, the shapes of EL spectra at various voltages remain unchanged (Figure 1(f)), suggesting good colour stability with the devices. The half-lifetime (*T*_50_) reaches 41 min at a constant current density of 3 mA cm^−2^, and the shapes of EL spectra are consistent during the lifetime measurement (Figure S2(b-c)). We also note that the shape of EL spectra remains unchanged during the 40 s operation time at a high current density of 100 mA cm^−2^ (Figure S2(d)). In contrast, the LED based on the control CsPb(Br_0.65_Cl_0.35_)_3_ film without additives shows very poor performance and spectral instability. It presents a low peak EQE of 0.07% and a luminance of ~100 cd m^−2^ at a voltage of 5 V (Figures 1(d) and 1(e)). The *T*_50_ is only 3 min at an initial luminance of 20 cd m^−2^, and an obvious shoulder appears in EL spectra after being biased for 1 min (Figure S2(e)), indicating a typical phase segregation of mixed-halide perovskites [7, 18]. By adding a single additive, Tween or TPPB, the device performance can also be improved significantly, although it remains less good than the performance of the Tween-TPPB device (Figure S2(f-h)).

We find that the device EL performance is consistent with the PL properties of the perovskite films. Figure 1(c) shows that the perovskite film with Tween and TPPB has a more symmetrical and narrow PL spectrum, reduced to 17 nm compared to the 31 nm of the control CsPb(Br_0.65_Cl_0.35_)_3_ film. The absorption spectra correspond very well with the PL spectra, showing a sharp optical absorption edge in the Tween-TPPB-based film, which is different from the broad absorption tail in the control CsPb(Br_0.65_Cl_0.35_)_3_ film (Figure S3(a)). We note that there is a small redshift in both PL and absorption for the Tween-TPPB-based film. The above optical measurement result suggests a well-ordered structure in Tween-TPPB-based CsPb(Br_0.65_Cl_0.35_)_3_, which is critical to achieve excellent electronic properties of the perovskite film [21, 22]. The transient PL measurements show that the PL lifetime (1/e of the initial value) of Tween-TPPB-based perovskites is 5 ns under a low fluence of 0.5 nJ cm^−2^ compared to the 2 ns of the control film, indicating reduced trap densities (Figure S3(b)). We then measure the PL emission of the films annealed under different temperatures. The control CsPb(Br_0.65_Cl_0.35_)_3_ film shows poor thermal stability (Figure S4). As the temperature is increased from 110 to 130°C, its PL peak is redshifted from 462 to 469 nm. In contrast, the PL spectra of the Tween-TPPB-based CsPb(Br_0.65_Cl_0.35_)_3_ film shows negligible change as the temperature increases.

### 2.2. Mechanism for the Suppressed Phase Segregation in Mixed-Halide Perovskites

**Figure 2:**
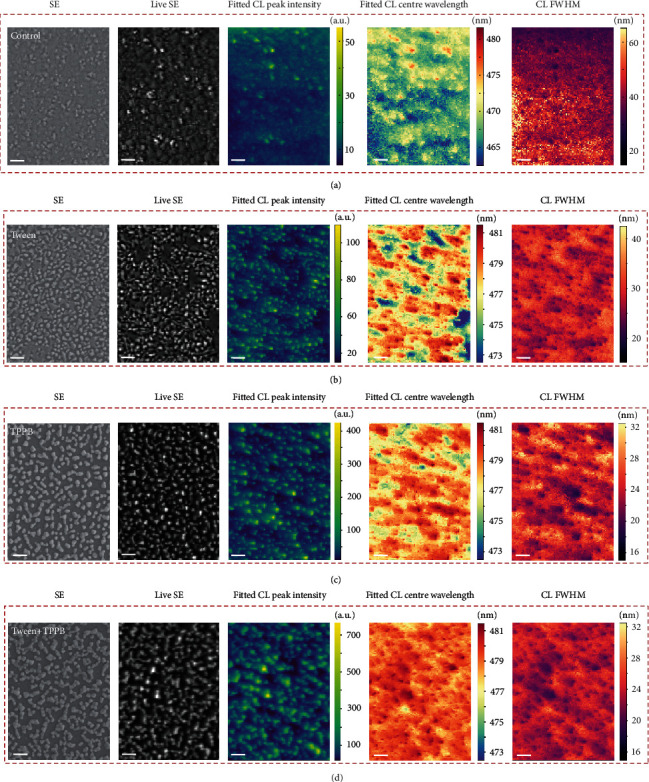
SEM and cathodoluminescence characteristics of perovskite films with various additives. The scale bar represents 500 nm. (a–d) SEM image, live SE image and corresponding CL intensity, CL peak, and CL FWHM maps of perovskite films without additive (a), with Tween (b), with TPPB (c), and with Tween and TPPB (d).

To reveal the origin of the phase stability in the Tween-TPPB-based CsPb(Br_0.65_Cl_0.35_)_3_ film, we investigate the local properties of mixed-halide perovskites by using cathodoluminescence (CL) scanning electron microscope (SEM). The SEM image shows that there is a heterogeneous distribution of the control CsPb(Br_0.65_Cl_0.35_)_3_ film with two distinct types of clusters, one bulging and another flat (Figure 2(a)). The CL spectra are measured with the same excitation conditions for the four films, and we can see that the inclusion of additives significantly enhances the CL peak intensity. Comparing the SEM image and CL map (Figure 2(a)), we observe a significant variation of local emission in the film. The bulging clusters in the SEM image show a higher CL intensity compared to others. Accordingly, the flat clusters demonstrate a blue-shifted emission peak and a wider spectrum than the bulging clusters (Figure 2(a) and Figure S5(a)), indicating a Cl-rich phase in the flat regions. Therefore, the above observations suggest that there is Cl-Br-phase segregation already present in the as-deposited mixed-halide perovskite film. Under electrical stress, the phase segregation can further develop and shift the electroluminescence spectrum to the red, as shown in Figure S2(e). We note that the inclusion of nonionic surfactant Tween molecules can facilitate the formation of bulging clusters and reduce the Cl-rich phase in the film (Figure 2(b)). From the local CL emission across the line scan and different spots (Figure S5(b)), we observe reduced variation of emission spectra. More significantly, the addition of TPPB assists the formation of uniformly discrete clusters, which rarely show the emission peak of the Cl-rich phase (Figure 2(c)). As a result, through the combined effect of Tween and TPPB, the phase separation in the CsPb(Br_0.65_Cl_0.35_)_3_ film is greatly suppressed, showing excellent homogeneity (Figure 2(d)). There is no obvious variation of the CL peak across the line scan as shown in Figure S5(d). Furthermore, the statistics of CL peaks of all measured pixels show that the emission peak mainly locates at 479 nm with a low standard deviation (SD) of 0.7 nm, while that of the control CsPb(Br_0.65_Cl_0.35_)_3_ film is 468 nm with a high SD of 2.4 nm (Figure S5(e)), which is consistent with the PL spectra (Figure 1(c)). We note that the consistency of the CL and PL spectra also indicates that the excitation dose during CL measurement did not cause degradation of our perovskites. Together with the EL measurement results, we believe that the macroscopically EL spectrum stability is governed by the microscopically halide homogeneity observed with the CL measurement.

**Figure 3:**
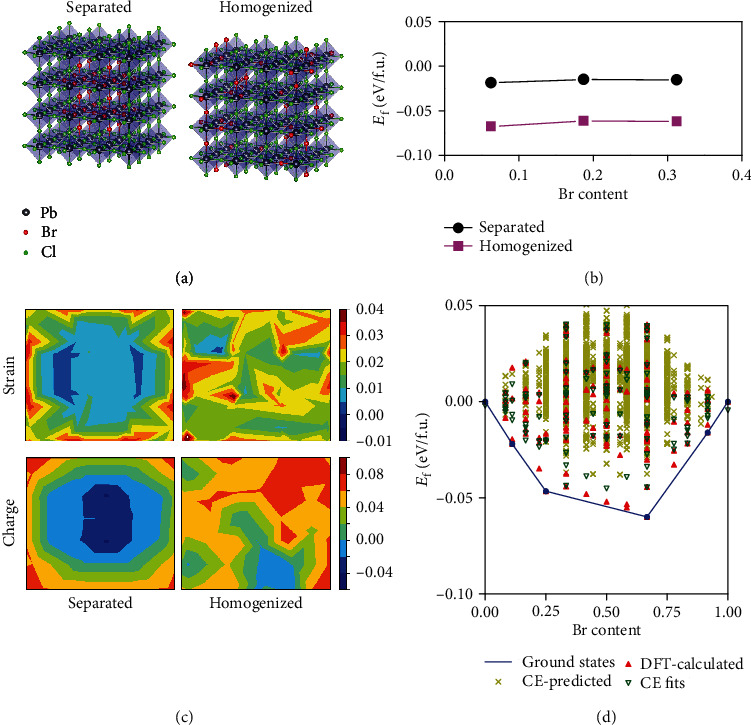
First-principle calculations of thermodynamic stability of CsPb(Br_1-*x*_Cl*_x_*)_3_ perovskites in phase-separated and phase-homogenized conditions. (a) Structures of phase-separated and phase-homogenized CsPb(Br_1-*x*_Cl*_x_*)_3_ perovskites involved in the calculations. The phase-homogenized structure corresponds to the random alloy. The cubic-phase perovskite structure is adopted, and for clarity only, the corner-sharing octahedral framework is shown. (b) Calculated formation energies (*E*_f_) with respect to decomposition into phase-pure CsPbBr_3_ and CsPbCl_3_. (c) Strain field (upper panels) and charge transfer (lower panels) distribution of one octahedral layer perpendicular to the [001] direction for the phase-separated and phase-homogenized cases. The strains are calculated in terms of Pb-Br/Cl bond lengths (see Materials and Methods). The charge transfer values (in unit charge) are evaluated by the difference in the charge at Br/Cl sites between CsPb(Br_1-*x*_Cl*_x_*)_3_ and CsPbBr_3_/CsPbCl_3_ (see Materials and Methods). The cases with the Br content of 0.1875 in (b) are selected for presentation. (d) Calculated formation energies of CsPb(Br_1-*x*_Cl*_x_*)_3_ perovskites in the whole component-variation range. The CsPb(Br*_x_*Cl_1-*x*_)_3_ structures were generated by the cluster expansion (CE) approach with the constraint of up to 20 atoms per unit cell (see Materials and Methods). The red triangles are the structures calculated by the density functional theory (DFT-calculated), and the blue line represents the stable ground states (ground states).

It is interesting to consider why halide separation in the as-made materials predisposes these films to undergo much more substantial halide segregation under electrical excitation in LEDs, whereas the more uniform initial halide distribution in the Tween-TPPB materials is much more resistant to subsequent halide segregation. The single additive perovskites show similar island-like morphology with the Tween-TPPB perovskites (Figure 2), while they have very different phase stability. So we believe that the morphology should not be the main reason of the enhanced phase stability. We then performed first-principle thermodynamic calculations of phase-heterogeneous and phase-homogeneous CsPb(Br_1-*x*_Cl*_x_*)_3_ perovskites (Figure 3(a)). The formation energies (*E*_f_) with respect to decomposition into phase-pure CsPbBr_3_ and CsPbCl_3_ were used to evaluate the thermodynamic stability. We find that the phase-homogenized case is energetically more favorable with an energy difference of ~60 meV/f.u. at various Br contents (Figure 3(b)), consistent with experimental results that the homogenous mixed-halide perovskite has better stability. We believe the phase-homogeneity-induced material stability originates from three facts: (i) The phase-homogenized case has a homogeneous and smaller-magnitude feature in the strain field distribution (Figure 3(c)), suggesting reduced strain energy. (ii) The phase-homogenized case shows homogeneous nature in the charge transfer from less-electronegative Br^−^ to more-electronegative Cl^−^ (Figure 3(c)). This more balanced charge distribution can minimize the emerged Coulomb interaction in the current ionic mixed-halide perovskite, leading to less energy gain [23]. In contrast, the phase-separated case with a large-area CsPbBr_3_/CsPbCl_3_ interface has substantial aggregation of strain and charge transfer, resulting in significantly increased stain energy and electric dipole-induced Coulomb energy. As shown in Figure 3(d), the homogenized CsPb(Br_1-*x*_Cl*_x_*)_3_ perovskites have much stable structures with respect to the phase-separation condition (i.e., with noticeable negative *E*_f_) through an expanded structure search in the whole component-variation range. The structures of the lowest-energy ground states show a more homogenized distribution of halogen atoms (Figure S6). (iii) Compared with the phase-separated case, the configuration entropy at the room temperature would result in further reduction of free energy and stabilize the homogeneous CsPb(Br_1-*x*_Cl*_x_*)_3_.

**Figure 4:**
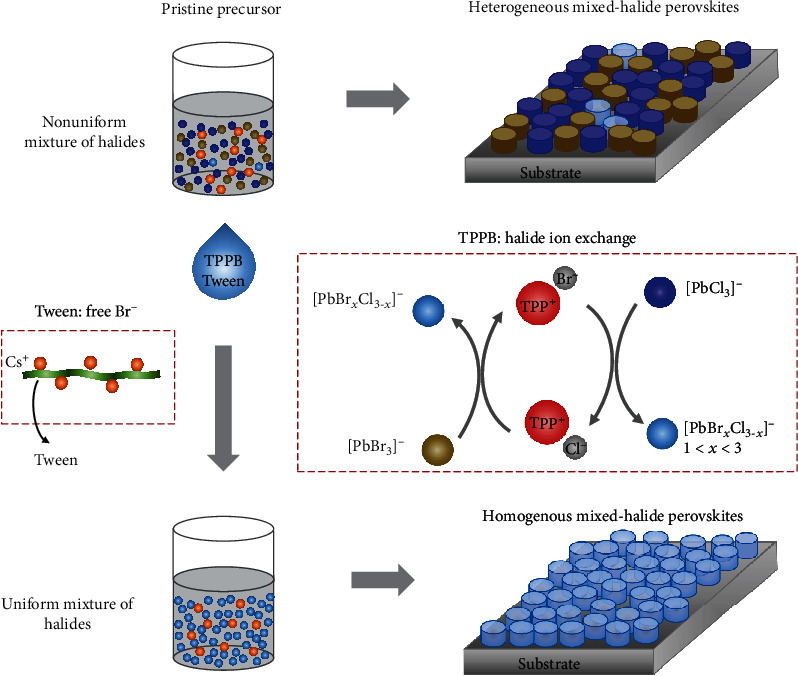
Schematic illustration of phase homogenization process through the assistance of Tween and TPPB.

We then explain the formation mechanism of the homogenous mixed-halide perovskites by the additives. The electrospray ionization time-of-flight mass spectrometry (ESI-TOF-MS) measurement shows that there is a main peak of [PbCl_3_]^−^ (Figure S7), suggesting that Br and Cl are not effectively mixed in the solution. As a result, the Br phase and Cl phase have a tendency to separately crystallize in different domains during the perovskite formation process, as we can observe in Figure 2(a). Importantly, the TPPB, which can work as a cationic surfactant in the precursor solution [24], induces the halide ion exchange to establish the chemical equilibrium (Figure 4). As shown in

(1) $$\begin{array}{c} x{\text{TPP}}^{+}{\text{Br}}^{‐}+{\left[{\text{PbCl}}_{3}\right]}^{‐}⇌\;{\left[{\text{PbCl}}_{3‐x}{\text{Br}}_{x}\right]}^{‐}+x{\text{TPP}}^{+}{\text{Cl}}^{‐}\\ \begin{array}{c} y{\text{TPP}}^{+}{\text{Cl}}^{‐}+{\left[{\text{PbBr}}_{3}\right]}^{‐}⇌{\left[{\text{PbCl}}_{y}{\text{Br}}_{3-y}\right]}^{‐}+y{\text{TPP}}^{+}{\text{Br}}^{‐} \end{array} \end{array}$$

the inclusion of TPPB can exchange a partial Cl ion in the Cl-rich complex. And the TPP^+^Cl^−^ also can promote the Br-rich complex to be a complex with more uniform Cl-Br distribution. This process can be verified by the ESI-TOF-MS measurement, which shows that after the addition of TPPB, multiple prominent peaks of [PbCl*_x_*Br_3-*x*_]^−^ (0 ≤ *x* ≤ 3) can be identified (Figure S7). The more uniform mixture of Cl-Br in the precursor results in a more homogenous mixed-halide phase during crystallization, as shown in Figure 2(c). Moreover, by Fourier transform infrared spectroscopy (FTIR) measurement, we observe that the Tween, which was used to tune the crystallization of perovskite [25], can weakly interact with Cs ion (Figure S8), further freeing the Br ion in the precursor and also facilitating to form a uniform Cl-Br distribution. Consequently, a homogeneous lead-halide complex can be formed in the precursor under the combined effect of Tween and TPPB, leading to crystallization of single-phase chlorine-bromine perovskites (Figure 2(d)). Figure 4 schematically presents this phase homogenization process in the mixed-halide perovskites. We note that the remaining Tween and TPPB mainly locate between the perovskite domains after the film formation, which can prevent the LED device from electrical short (Figure 1(d)) and enhance the light outcoupling efficiency [4]. However, when the additive ratio is further increased, the inferior charge transport induced by the organic additive would affect the EQE of the device (Figure S9).

**Figure 5:**
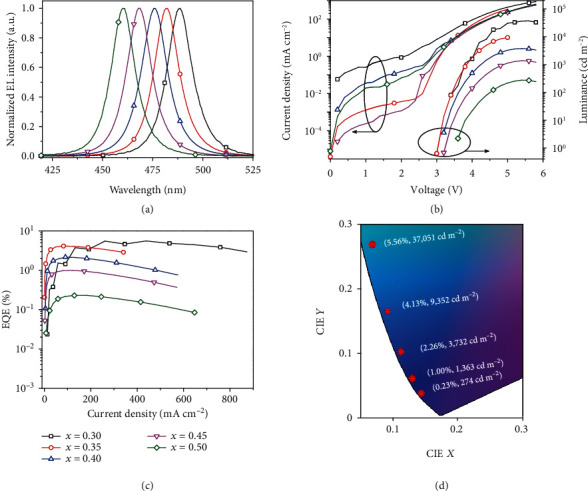
Characterization of perovskite LEDs based on CsPb(Br_1-*x*_Cl*_x_*)_3_ films, *x* = 0.30, 0.35, 0.40, 0.45, and 0.50: (a) normalized EL spectra; (b) current density and luminance versus voltage; (c) EQE versus current density; (d) corresponding CIE coordinates. The peak EQE and maximum luminance of LEDs are also presented.

Inspired by the excellent property of the Tween-TPPB-based CsPb(Br_0.65_Cl_0.35_)_3_ film, we further change the ratio of bromide and chloride in the precursor solution.  Figure 5 shows that we can tune the EL emission peaks from 489 to 460 nm as the Br : Cl ratio changes from 70 : 30 to 50 : 50. All the devices show narrow EL spectra with FWHM of ~15 nm, suggesting excellent homogeneity and colour purity. The emission of the sky blue CsPb(Br_0.7_Cl_0.3_)_3_ LED locates at a CIE coordinate of (0.068, 0.268) and reaches a peak luminous efficacy of 4.7 lm W^−1^ and a maximum luminance of 37,000 cd m^−2^, which is three times higher than pervious works (Table S1) [10, 12, 26]. The device has low efficiency roll-off and can maintain an EQE of 5.6% at a high luminance of 30,000 cd m^−2^ (corresponding to a large current density of 438 mA cm^−2^). Moreover, the deep blue CsPb(Br_0.55_Cl_0.45_)_3_ LED (CIE: 0.129, 0.061) reaches a peak luminous efficacy of 0.4 lm W^−1^ and a maximum luminance of 1,360 cd m^−2^, which is also the record luminance [27–29]. Importantly, all the devices exhibit good colour stability (Figure S10). We note that the device efficiency and brightness can be further enhanced in the future if the photoluminescence quantum efficiency (PLQE) of the Tween-TPPB-based CsPb(Br_0.65_Cl_0.35_)_3_ film can be improved from the current 20% (Figure S3(c)) with defect passivation approaches [4, 6].

## 3. Discussion

Phase segregation in mixed-halide perovskites has been a long-lasting issue in the community [7, 12]. By using high-resolution cathodoluminescence microscopy, we directly revealed that the phase segregation is caused by the intrinsic and microscopic phase heterogeneity in as-deposited mixed-halide perovskites, which resulted from a precursor solution with a nonuniform mixture of halides. We demonstrated that cationic surfactants can effectively overcome this issue by homogenizing the halides in the precursor solution. Efficient deep blue, blue, and sky blue EL with very high brightness has been realized with this simple strategy. We believe our finding is important not only for perovskite LEDs but also for the perovskite solar cell community, where the bandgaps of the perovskites need to be carefully tuned by mixing halides in order to maximize the photovoltaic efficiency with tandem structures [30].

## 4. Materials and Methods

### 4.1. Material Preparation

The precursor solutions of perovskites were prepared by dissolving CsBr, PbBr_2_, and PbCl_2_ with a molar ratio of 1.5 : 0.39 : 0.61 in a mixed solution of DMSO and H_2_O (volume ratio, 0.92 : 0.08) at a concentration of 10 wt%. The Tween refers to Tween 20 in this work. For the Tween-based perovskite, Tween was kept 1.6 mM in the precursor solution. For the TPPB-based perovskite, the ratio of TPPB, CsBr, PbBr_2_, and PbCl_2_ was 0.075 : 1.5 : 0.37 : 0.63 to keep the Br : Cl to 65 : 35. For perovskite-incorporated Tween and TPPB, the molar ratios of CsBr, PbBr_2_, and PbCl_2_ were changed to 1.5 : 0.46 : 0.54 (10 wt%), 1.5 : 0.37 : 0.63 (10 wt%), 1.5 : 0.29 : 0.71 (8.3 wt%), 1.5 : 0.20 : 0.80 (7.1 wt%), and 1.5 : 0.11 : 0.89 (6.3 wt%) to control the Br : Cl to 70 : 30, 65 : 35, 60 : 40, 55 : 45, and 50 : 50, respectively. All precursor solutions were stirred at 90°C for 4 h in a glovebox.

### 4.2. Device Fabrication

The PEDOT:PSS (Clevios P VP 4083) was spin-coated onto ITO glass at 5000 rpm for 50 s and annealed at 150°C for 10 min. Then, the perovskite solution was spin-coated onto the PEDOT:PSS layer at 6000 rpm for 45 s and annealed at 130°C for 20 min in the glovebox. Finally, TmPyPB, LiF, and Al were sequentially thermally evaporated. The device area was 3 mm^2^.

### 4.3. Device Characterization

All perovskite LEDs were characterized using an integration sphere (FOIS-1) coupled with a QE65 Pro spectrometer system [30, 31]. The LEDs were swept with a scanning rate of 0.2 V s^−1^. The characterization of the device stability was carried out in a glovebox using the same testing system.

### 4.4. Film Characterization

The film thickness is measured by using a P-7 Stylus Profiler (KLA-Tencor). Absorption and PL spectra were measured using a spectrophotometer with an integrating sphere and a QE65 Pro spectrometer with a 375 nm CW laser as an excitation source. XRD data were collected by using a Bruker D8 Advance. The PLQE of perovskite films was measured in an integrating sphere based on a three-step technique using a 375 nm CW laser as an excitation source [32]. The time-resolved PL measurements were performed by a combination of a TimeHarp 260 PICO module (PicoQuant), an iHR320 spectrometer (Horiba), and COUNT-100T-FC single photon counting modules (Laser Components GmbH), and the quartz/PEDOT:PSS/perovskite samples were excited by a 375 nm pulsed laser at a repetition rate of 2 MHz. Mass spectra were recorded with an Agilent 6230 TOF LC/MS. FTIR spectra were recorded by using a Thermo Scientific Nicolet iS50 with reflectance mode. The films were deposited on Au-coated glass substrates. SEM images of perovskite films on ITO/PEDOT:PSS substrates were obtained using a JEOL5 JSM-7800F SEM at 3 kV accelerating voltage. CL hyperspectral mapping of perovskite films on ITO/PEDOT:PSS substrates was performed in an Attolight Allalin 4027 Chronos dedicated CL-SEM. CL spectra and images were recorded by using an iHR320 spectrometer with a focal length of 320 mm with a 150 l/mm grating blazed at 500 nm, a 700 *μ*m entrance slit, and an Andor 1024 px charged coupled device. All CL spectra were recorded with an acceleration energy of 6 keV and a beam current of 1 nA. The acceleration energy corresponds to an interaction volume of xyz (performed with a Monte Carlo Simulation using actual device structure and densities) according to xzy (free to download/use: https://www.gel.usherbrooke.ca/casino/What.html). Here, the effective CL probe diameter at the signal decreasing to 10% is ~80 nm, and the pixel size is 39 nm × 39 nm.

### 4.5. First-Principle Calculations

Energetic calculations were carried out within the framework of the density functional theory using plane-wave pseudopotential method as implemented in the Vienna Ab initio Simulation Package [33, 34]. The electron-ion interactions were described by using the projected augmented wave pseudopotentials. The 5s^2^5p^6^6s^1^ (Cs), 6s^2^6p^2^ (Pb), 4s^2^4p^5^ (Br), and 3s^2^3p^5^ (Cl) were treated explicitly as valence electrons. We used the generalized gradient approximation as the exchange correlation functional [35]. The CsPbBr_3_/CsPbCl_3_ core-shell-like structures and the random CsPb(Br*_x_*Cl_1-*x*_)_3_ alloys were constructed to simulate the phase-separated and phase-homogenized cases of CsPb(Br*_x_*Cl_1-*x*_)_3_ perovskites. The 4 × 4 × 4 supercell of the cubic-phase perovskite structure was adopted for these calculations. The Br content *x* of 0.0625, 0.1875, and 0.3125 were selected. The random CsPb(Br*_x_*Cl_1-*x*_)_3_ alloys were generated by the Special Quasirandom Structure (SQS) method [36, 37], which can produce the best approximated periodic ordered structure to mimic the actual disordered state. Structure optimization (including lattice parameters and internal atomic positions) was performed using the conjugate gradient technique [38] until the energies converged to 10^−5^ eV. A kinetic energy cutoff of 340 eV was used for wave-function expansion and a *Γ*-point-only *k*-point mesh was used for Brillouin zone integration. The structure searches of CsPb(Br*_x_*Cl_1-*x*_)_3_ perovskites in the whole component-variation range were performed by using the cluster expansion approach as implemented in the ATAT code [39]. A total of 140 structures with up to 20 atoms per unit cell of CsPb(Br*_x_*Cl_1-*x*_)_3_ perovskites were calculated. In these calculations, the *k*-point mesh with a grid spacing of 2*π* × 0.03 Å^−1^ was used, and the structure optimization was done with the residual forces on the atoms converged to below 0.01 eV/Å. The local strains were evaluated in terms of the difference in the Pb-Br/Cl bond lengths between the CsPb(Br*_x_*Cl_1-*x*_)_3_ perovskites and the phase-pure CsPbBr_3_/CsPbCl_3_, e.g., *ε* = (*l*_2_ − *l*_1_)/*l*_1_, where *ε* is the local strain, *l*_2_ is the bond length of CsPb(Br*_x_*Cl_1-*x*_)_3_ and *l*_1_ is the bond length of CsPbBr_3_/CsPbCl_3_. Similar to the local strain calculations, the charge transfer values (in unit charge) are evaluated by the difference in the charge at Br/Cl sites between CsPb(Br*_x_*Cl_1-*x*_)_3_ and CsPbBr_3_/CsPbCl_3_. The charge at Br/Cl sites was obtained based on Bader charge analysis [40].

## Supplementary Materials

Supplementary Materials Figure S1: PL spectra of perovskite films fabricated from precursor solutions with or without H_2_O. Figure S2: characterization of perovskite LEDs with various additives. Figure S3: optical characterization of CsPb(Br_0.65_Cl_0.35_)_3_ films. Figure S4: PL spectra of perovskite films annealed at different temperatures for 20 min. Figure S5: CL spectra of line scans in live SEM images and statistics of CL peaks. Figure S6: calculated formation energies of CsPb(Br_*x*_Cl_1-*x*_)_3_ perovskites in the whole component-variation range (upper panel). Figure S7: ESI-TOF-MS spectra. Figure S8: FTIR spectra of Tween and Tween:CsBr films. Figure S9: characterizations of the perovskite LEDs with various Tween and TPPB ratios. Figure S10: EL spectra of perovskite LEDs under various bias voltages. Table S1: comparison of our devices with reported spectrally stable blue perovskite LEDs.
