# Supplementary material for: Halide Homogenization for High-Performance Blue Perovskite Electroluminescence
Source: Research (Wash D C). 2020 Dec 24;2020:9017871. doi: 10.34133/2020/9017871 (PMC7877380; doi:10.34133/2020/9017871)
Supplement: Supplementary Materials — Figure S1: PL spectra of perovskite films fabricated from precursor solutions with or without H2O. Figure S2: characterization of perovskite LEDs with various additives. Figure S3: optical characterization of CsPb(Br0.65Cl0.35)3 films. Figure S4: PL spectra of perovskite films annealed at different temperatures for 20 min. Figure S5: CL spectra of line scans in live SEM images and statistics of CL peaks. Figure S6: calculated formation energies of CsPb(BrxCl1-x)3 perovskites in the whole component-variation range (upper panel). Figure S7: ESI-TOF-MS spectra. Figure S8: FTIR spectra of Tween and Tween:CsBr films. Figure S9: characterizations of the perovskite LEDs with various Tween and TPPB ratios. Figure S10: EL spectra of perovskite LEDs under various bias voltages. Table S1: comparison of our devices with reported spectrally stable blue perovskite LEDs. [file 9017871.f1.docx]

**Supplementary Information**

Halides Homogenization for High-Performance Blue Perovskite Electroluminescence

Lu Cheng^1^, Chang Yi^1^, Yunfang Tong^1^, Lin Zhu^1^, Gunnar Kusch^2^, Xiaoyu Wang^3^, Xinjiang Wang^3^, Tao Jiang^1^, Hao Zhang^1^, Ju Zhang^1^, Chen Xue^4^, Hong Chen^1^, Wenjie Xu^1^, Dawei Liu^1^, Rachel A. Oliver^2^, Richard H. Friend^5^, Lijun Zhang^3*^, Nana Wang^1,5*^, Wei Huang^1,4*^ and Jianpu Wang^1*^

^1^Key Laboratory of Flexible Electronics (KLOFE) & Institute of Advanced Materials (IAM), Nanjing Tech University (NanjingTech), 30 South Puzhu Road, Nanjing 211816, China.

^2^Department of Materials Science and Metallurgy, University of Cambridge, 27 Charles Babbage Road, Cambridge CB3 0FS, UK.

^3^State Key Laboratory of Integrated Optoelectronics, Key Laboratory of Automobile Materials of MOE and College of Materials Science and Engineering, Jilin University, Changchun 130012, China.

^4^Frontiers Science Center for Flexible Electronics (FSCFE) & Shaanxi Institute of Flexible Electronics (SIFE), Northwestern Polytechnical University (NPU), 127 West Youyi Road, Xi'an 710072, China

^5^Cavendish Laboratory, University of Cambridge, JJ Thomson Avenue, Cambridge CB3 0HE, UK.

Correspondence should be addressed to Prof. Jianpu Wang (iamjpwang@njtech.edu.cn); Prof. Wei Huang (iamwhuang@nwpu.edu.cn); Prof. Nana Wang (iamnnwang@njtech.edu.cn); Prof. Lijun Zhang (lijun_zhang@jlu.edu.cn).


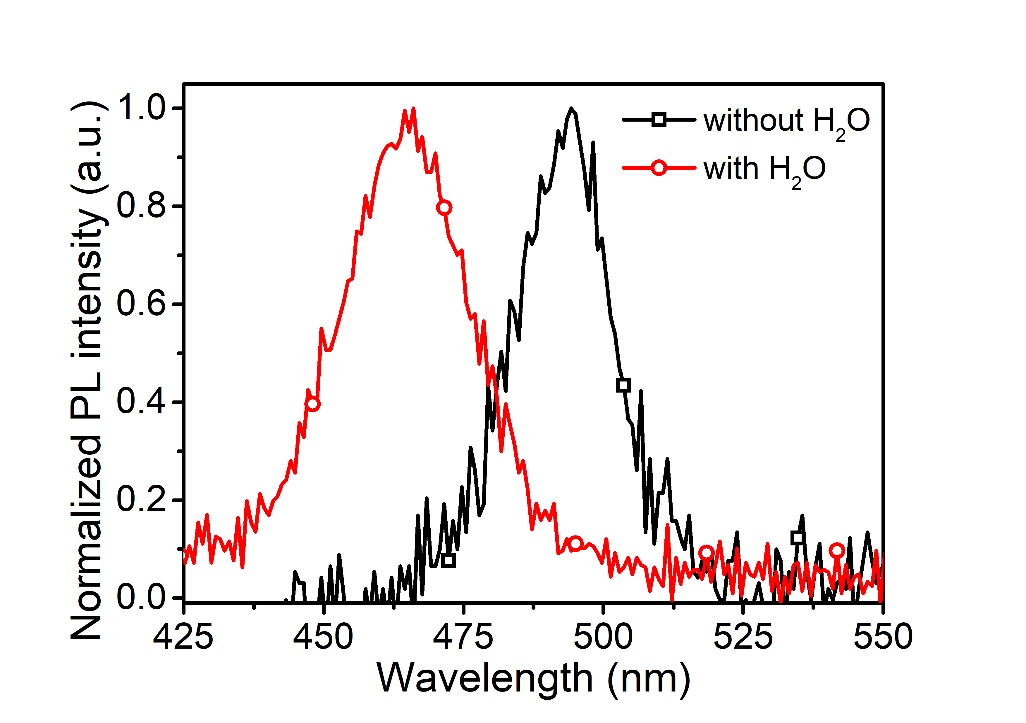


**Figure S1. PL spectra of perovskite films fabricated from precursor solutions with or without H_2_O.**
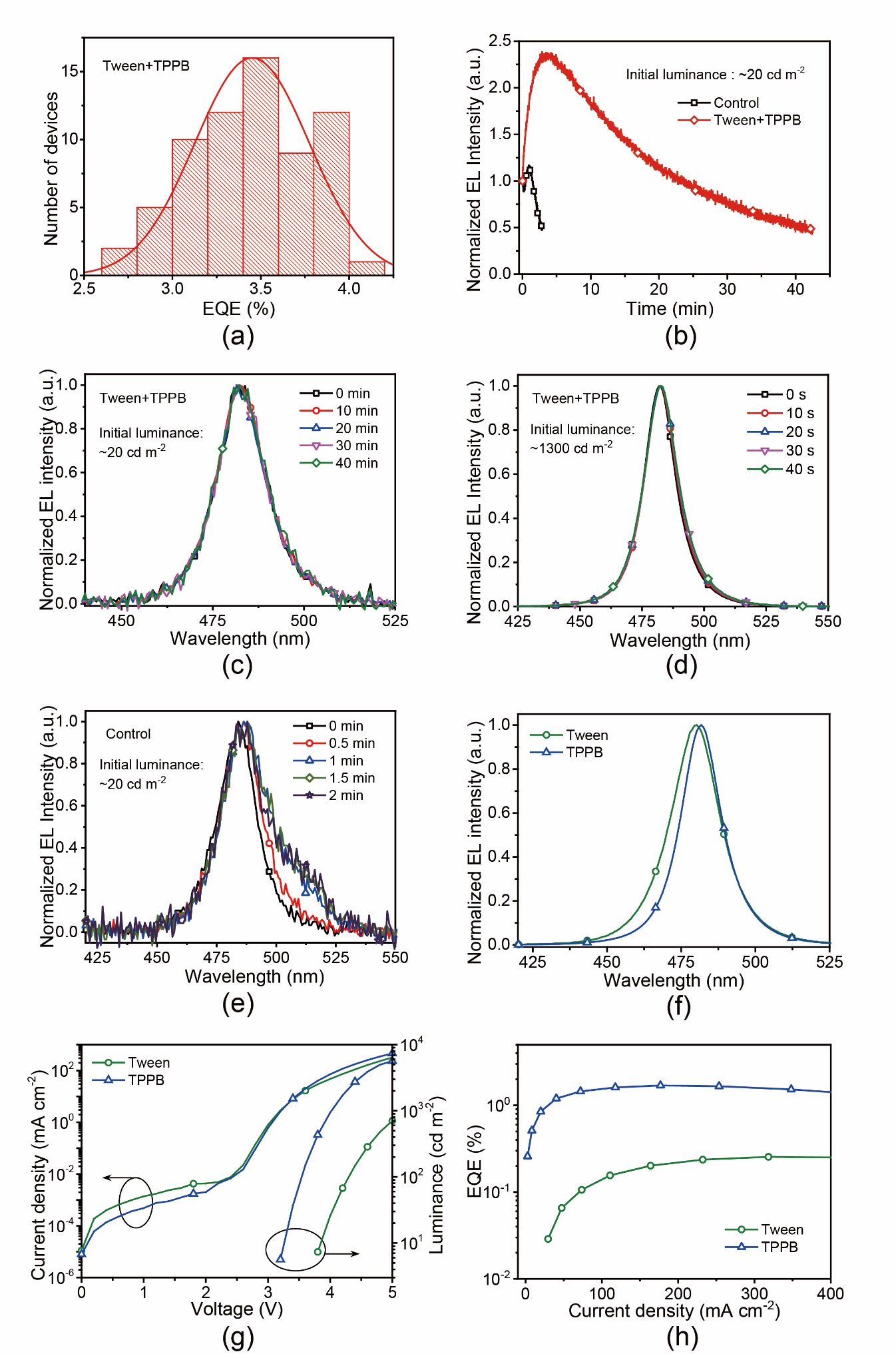


**Figure S2. Characterization of perovskite LEDs with various additives.** (a) Histogram of peak EQEs of 67 Tween-TPPB based devices. (b) Stability of devices measured at an initial luminance of ~20 cd m^-2^. The control CsPb(Br_0.65_Cl_0.35_)_3_ and Tween-TPPB based devices were measured at constant current density of 150 and 3 mA cm^-2^, respectively. (c-d) EL spectra upon various operation time of Tween-TPPB based device at constant current densities of 3 mA cm^-2^ (c) and 100 mA cm^-2^ (d). (e) EL spectra at various operation times of CsPb(Br_0.65_Cl_0.35_)_3_ device at a constant current density of 3 mA cm^-2^. (f) EL spectra. (g) Current density and luminance versus voltage. (h) EQE versus current density.


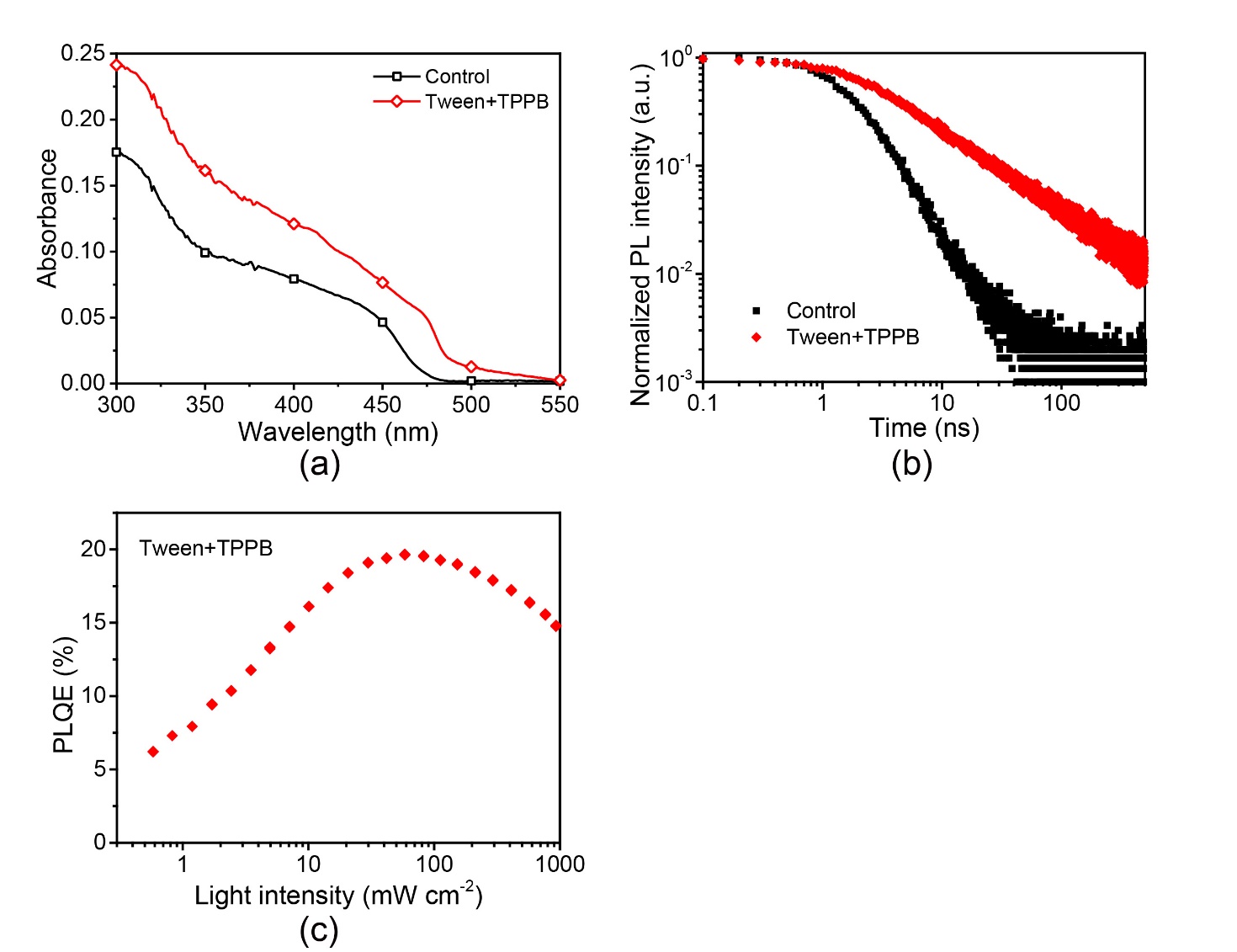


**Figure S3. Optical characterization of CsPb(Br_0.65_Cl_0.35_)_3_ films.** (a) Absorption spectra. (b) Time-resolved PL for the films under a fluence of 0.5 nJ cm^-2^. (c) Excitation-intensity-dependent PLQE.


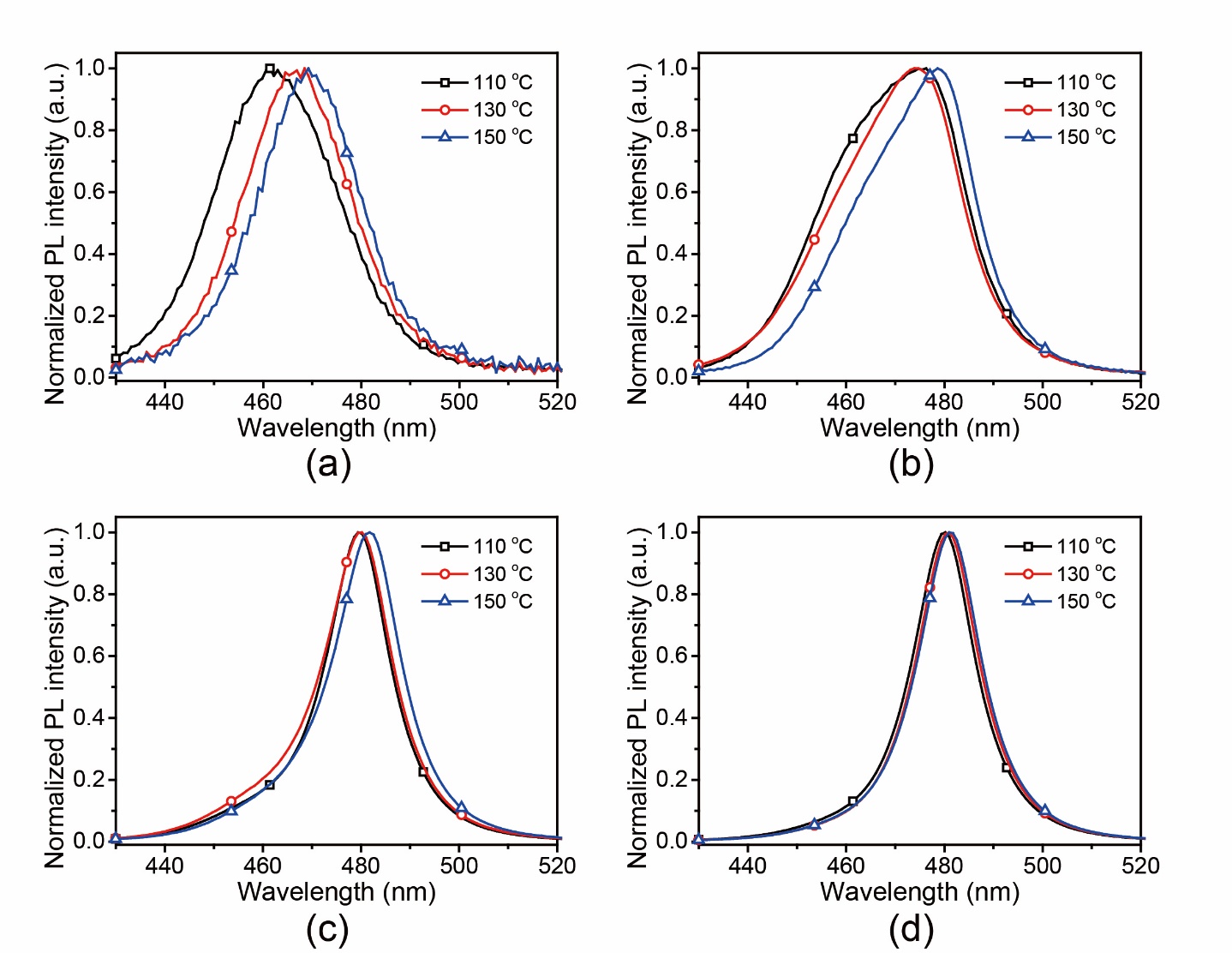


**Figure S4. PL spectra of perovskite films annealed at different temperatures for 20 min.** (a) without additive. (b) with Tween. (c) with TPPB. (d) with Tween and TPPB.


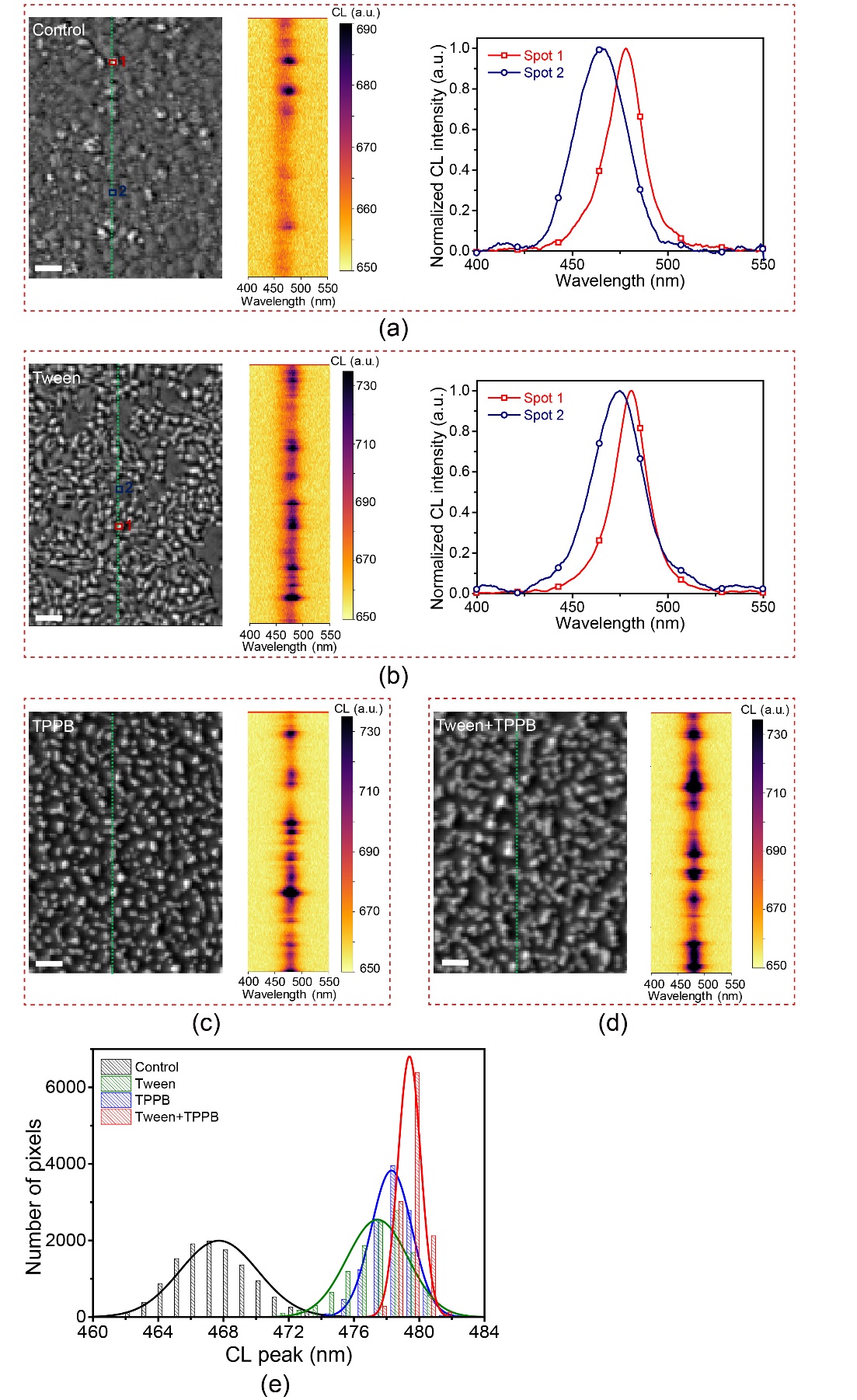


**Figure S5. CL spectra of line scans in Live SEM images and statistics of CL peaks.** The scale bar represents 500 nm. (a-d) CL spectra of line scans in films without additive (a), with Tween (b), with TPPB (c), with Tween and TPPB (d). It shows that as the inclusion of additives, the variation of CL peak and FWHM is reduced. (e) Statistics of CL peak of measured pixels. The SD values of films without additive, with Tween, with TPPB and with Tween-TPPB are 2.4, 1.9, 1.2, 0.7 nm, respectively. This indicates the local emission peak in Tween-TPPB based film locates in a narrowest range.

**
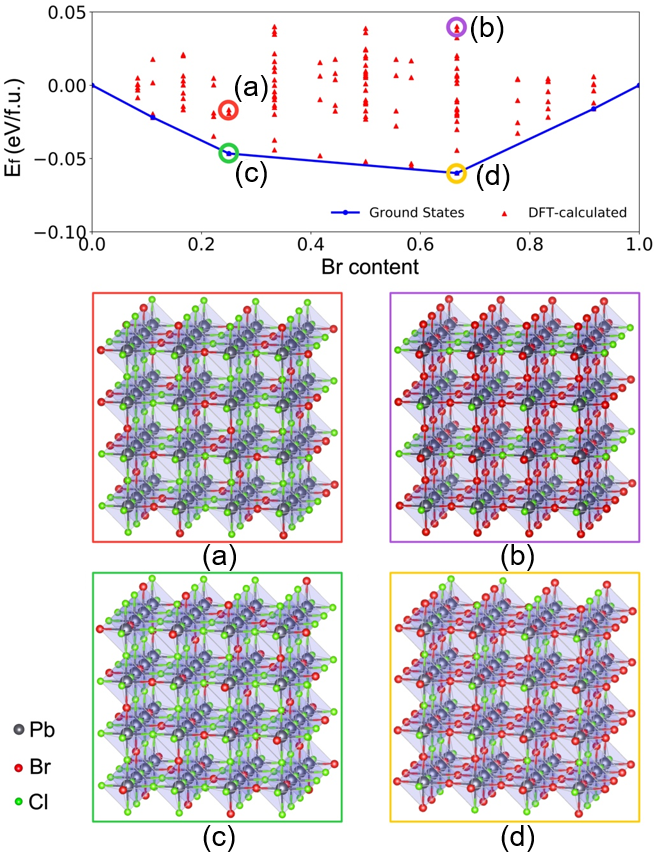
**

**Figure S6. Calculated formation energies of CsPb(Br*_x_*Cl_1-_*_x_*)_3_ perovskites in the whole component-variation range (upper panel).** Only the structures calculated by the density functional theory (DFT-calculated) were shown and the stable ground states (Ground States) are indicated. At the Br contents of 0.25 and 0.667, two stable ground states were found. (a,c) The structures corresponding to the points marked at the Br content of 0.25 in the upper panel. (b,d) The structures corresponding to the points marked at the Br content of 0.667 in the upper panel. For clarity only corner-sharing octahedral framework is shown. It indicates that at both Br contents, the lowest-energy ground states show a more homogenized distribution of halogen atoms, whereas the highest-energy structures show aggregation tendency of halogen atoms viewed along particular directions.

**
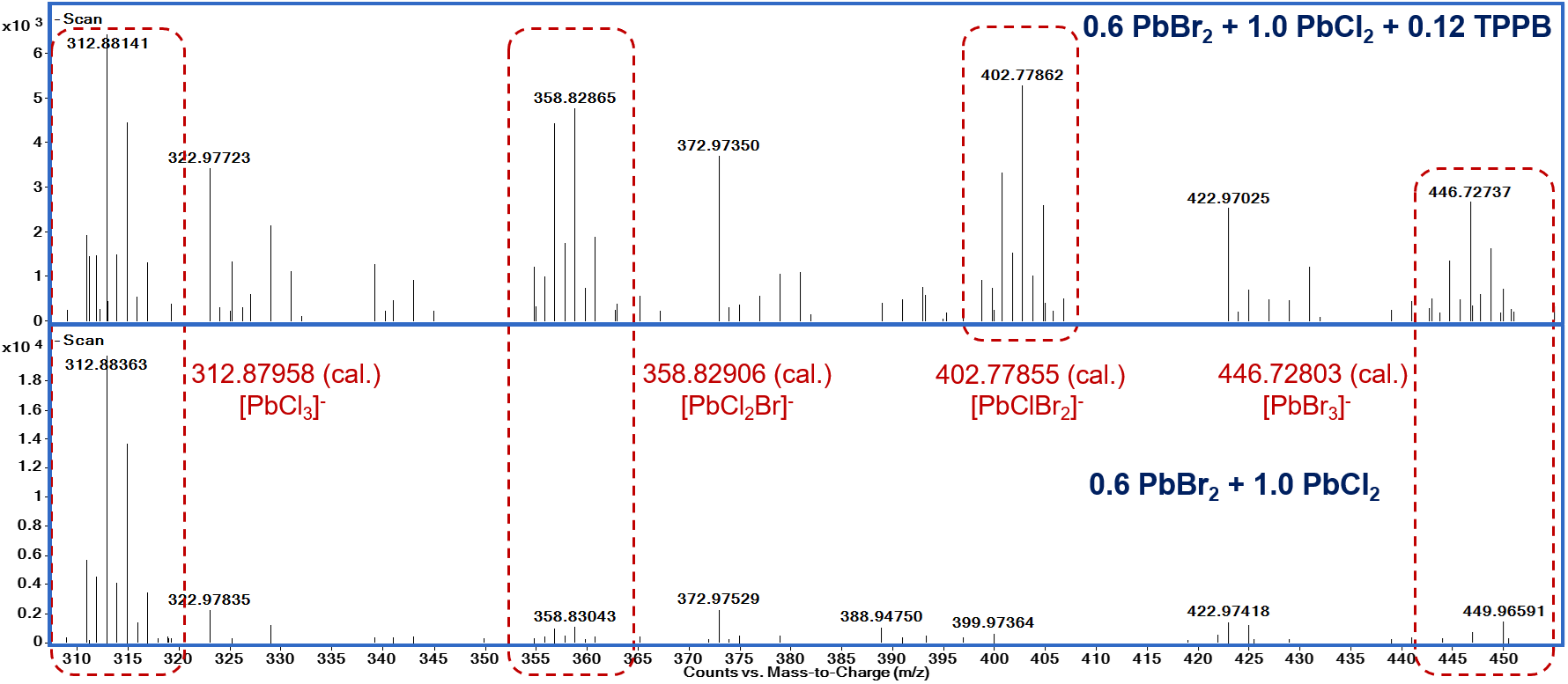
**

**Figure S7. ESI-TOF-MS spectra.** The PbBr_2_/PbCl_2_ (0.6/1.0) solution shows a main [PbCl_3_]^–^ peak and weak [PbCl_2_Br]^–^, [PbBr_3_]^–^ peaks. As the inclusion of TPPB, there appears multiple prominent peaks at *m/z* = 312.88141, 358.82865, 402.77862, 446.72737, corresponding to [PbCl_3_]^–^, [PbCl_2_Br]^–^, [PbClBr_2_]^–^ and [PbBr_3_]^–^. All the assigned peaks were in good agreement with the calculated theoretical distribution, indicating TPPB can effectively facilitate the halide ion exchange in the precursor solution.

**
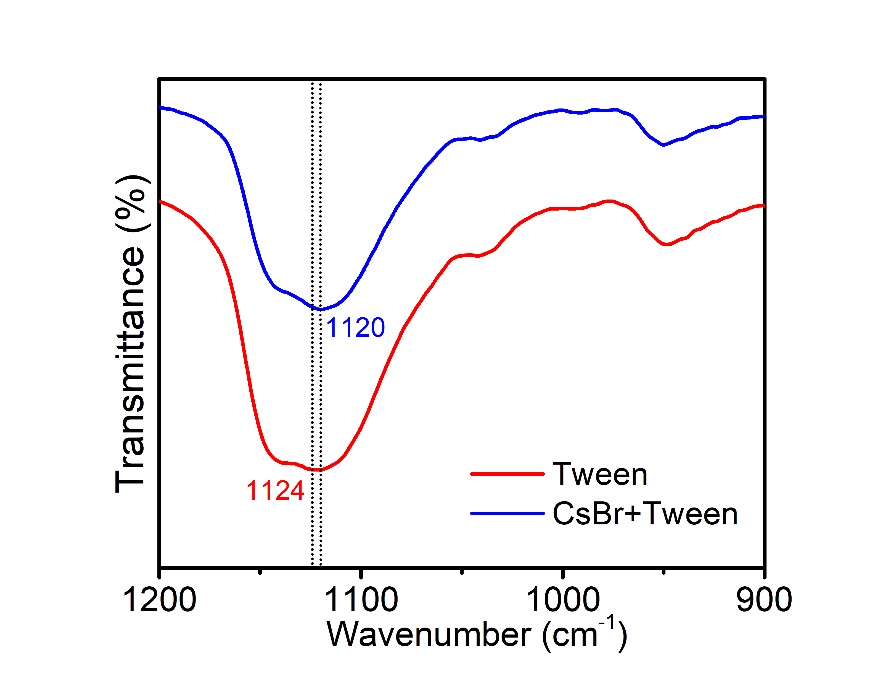
**

**Figure S8. FTIR spectra of Tween and Tween:CsBr films.** The peak at 1124 cm^-1^ associated with the C-O-C stretching vibration in Tween is shifted to 1120 cm^-1^ upon the addition of CsBr.


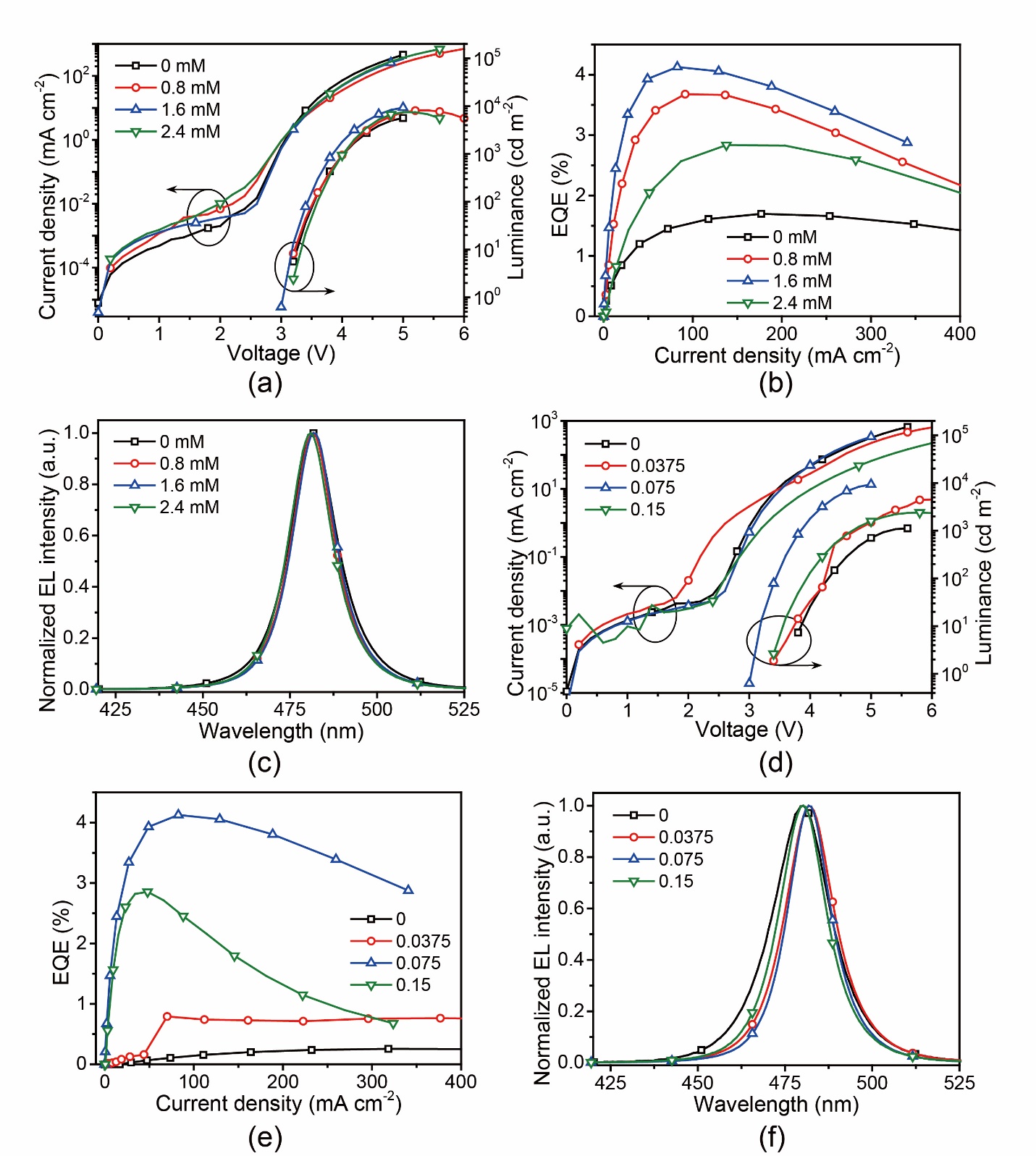


**Figure S9. Characterizations of the perovskite LEDs with various Tween and TPPB ratios.** (a,d) Dependence of current density and luminance of devices with Tween (a) and TPPB (d). (b,e) EQE versus current density of devices with Tween (b) and TPPB (e). (c,f) EL spectra of devices with Tween (c) and TPPB (f).


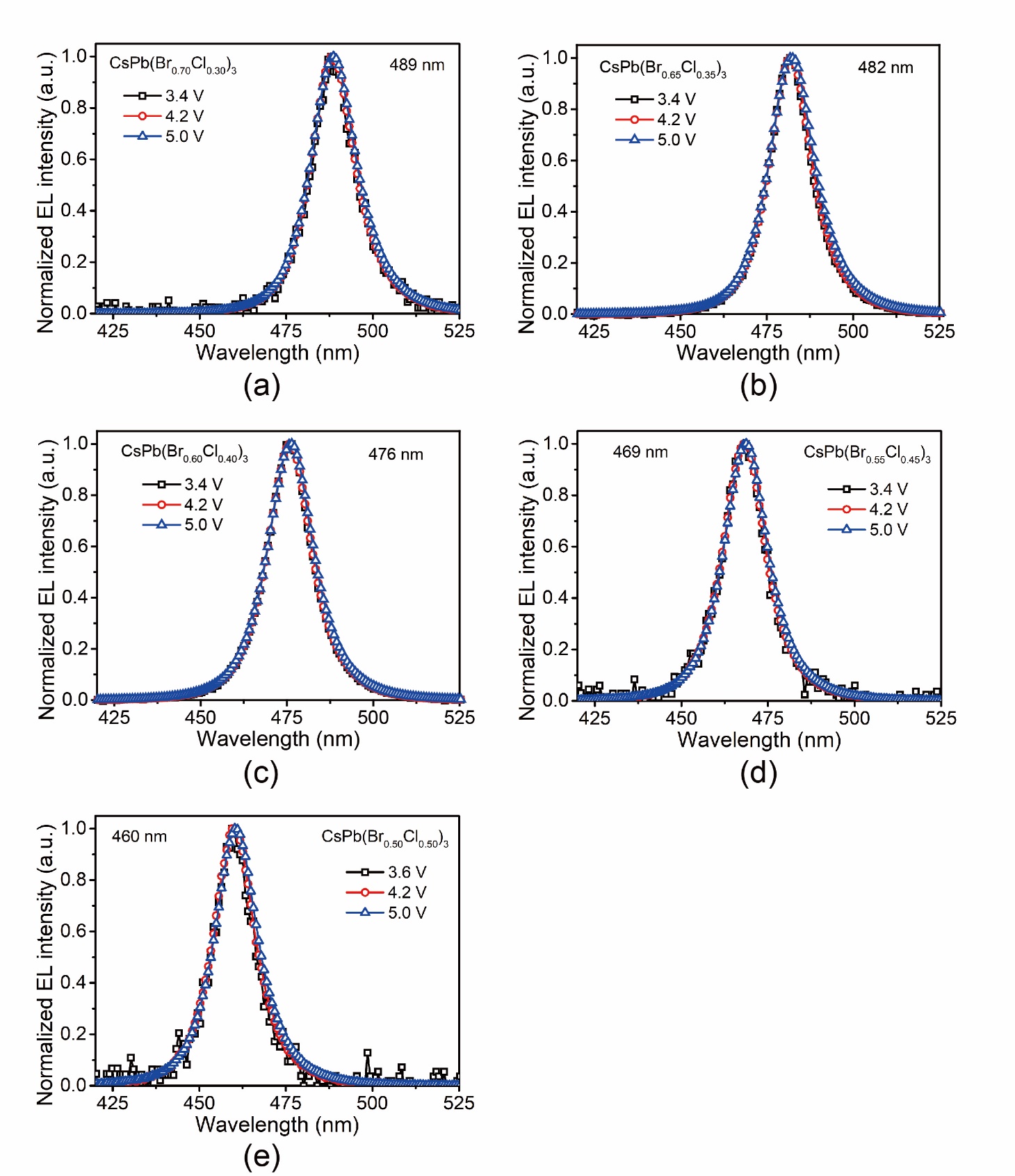


**Figure S10. EL spectra of perovskite LEDs under various bias voltages.** (a) CsPb(Br_0.70_Cl_0.30_)_3_ device. (b) CsPb(Br_0.65_Cl_0.35_)_3_ device. (c) CsPb(Br_0.60_Cl_0.40_)_3_ device. (d) CsPb(Br_0.55_Cl_0.45_)_3_ device. (e) CsPb(Br_0.50_Cl_0.50_)_3_ device.**Table S1. Comparison of our devices with reported spectral-stable blue perovskite LEDs.**

|  | EL peak  (nm) | FWHM  (nm) | CIE | Maximum luminance  (cd m^-2^) | Peak EQE  (%) | Luminance at peak EQE  (cd m^-2^) | Luminous efficacy (lm W^-1^) | Reference |
| --- | --- | --- | --- | --- | --- | --- | --- | --- |
| Sky blue | 475 | 28 | (0.138, 0.268) | 3,567 | 1.7 | - | 6.6 | (*12*) |
|  | 489 | 15 | (0.068, 0.268) | 37,051 | 5.56 | 30,562 | 4.7 | This work |
|  | 484 | 18 | (0.08, 0.21) | 9,243 | 1.66 | ~7,550^*^ | - | (*26*) |
|  | 485 | ~23^*^ | (0.09, 0.19) | 9,040 | 11.0 | 955 | - | (*10*) |
|  | 483 | ~26^*^ | (0.094, 0.184) | 700 | 9.5 | ~50^*^ | - | (*9*) |
| Blue | 480 | 21 | (0.102, 0.178) | 3,780 | 5.7 | ~1,268^*^ | - | (*18*) |
|  | 482 | 15 | (0.091, 0.165) | 9,352 | 4.13 | 3,180 | 2.9 | This work |
|  | 477 | ~23^*^ | (0.10, 0.13) | 5,619 | 4.8 | 2,583 | - | (*10*) |
|  | 476 | 15 | (0.112, 0.103) | 3,732 | 2.16 | 1,439 | 1.1 | This work |
|  | 474 | ~27^*^ | (0.122, 0.108)^†^ | 120 | 4.0 | ~15^*^ | - | (*9*) |
|  | 471 | 17 | (0.129, 0.087) | 465 | 6.3 | ~11^*^ | - | (*19*) |
| Deep blue | 469 | 15 | (0.129, 0.061) | 1,363 | 1.00 | 666 | 0.4 | This work |
|  | 465 | 25 | (0.145, 0.05) | 211 | 2.6 | ~5^*^ | - | (*27*) |
|  | 460 | 15 | (0.143, 0.038) | 274 | 0.23 | 171 | 0.06 | This work |
|  | 452 | 16 | (0.152, 0.023)^*^ | 150 | 0.08 | - | 0.02 | (*29*) |

^*^ These data were estimated from the figures in the references.

^†^ These data were estimated from the EL spectra.
